# Sex differences in hemodynamics and outcomes after transcatheter aortic valve replacement

**DOI:** 10.1007/s00392-025-02794-2

**Published:** 2025-11-20

**Authors:** Henning Guthoff, Mohamed Abdel-Waha, Won-Keun Kim, Hendrik Wienemann, Jasmin Shamekhi, Clemens Eckel, Ina von der Heide, Verena Veulemans, Martin Landt, Jury Schewel, Nicolas M. Van Mieghem, Rik Adrichem, Stefan Toggweiler, Tobias Rheude, Sascha Macherey-Meyer, Sabine Bleiziffer, Baravan Al-Kassou, Stephan Nienaber, Jan Wrobel, Ines Richter, Helge Möllmann, Matthias Renker, Efstratios Charitos, Niklas Schofer, Tobias Zeus, Tobias Schmidt, Philipp von Stein, Holger Thiele, Guy Witberg, Matti Adam, Stephan Baldus, Tanja K. Rudolph, Victor Mauri

**Affiliations:** 1 Clinic III for Internal Medicine, University of Cologne, Faculty of Medicine and University Hospital Cologne, Cologne, Germany; 2 Center for Cardiovascular Medicine ABCD, Aachen, Bonn, Cologne, Düsseldorf, Germany; 3 Department of Cardiology, Heart Center Leipzig at University of Leipzig, Leipzig, Germany; 4 Department of Cardiology, Kerckhoff Heart Center, Bad Nauheim, Germany; 5 Department of Cardiac Surgery, Kerckhoff Heart Center, Bad Nauheim, Germany; 6 Department of Cardiology, Justus-Liebig University of Giessen, Giessen, Germany; 7 German Center for Cardiovascular Research (DZHK), Partner Site RhineMain, Bad Nauheim, Germany; 8 Heart Center, Department of Medicine II, University Hospital Bonn, Bonn, Germany; 9 Department of Cardiology, St. Johannes Hospital, Dortmund, Germany; 10 Department of Cardiology, University Heart & Vascular Center Hamburg and the German Center for Cardiovascular Research (DZHK), Partner Site Hamburg/Lübeck/Kiel, Hamburg, Germany; 11 Department of Cardiology, Helios Heart Centre Siegburg, Siegburg, Germany; 12 Heart Center, Segeberger Kliniken, Bad Segeberg, Germany; 13 Department of Cardiology, Marienkrankenhaus Hamburg, Hamburg, Germany; 14 Department of Cardiology, Asklepios Klinik St. Georg, Hamburg, Germany; 15 Department of Interventional Cardiology, Thoraxcenter, Erasmus University Medical Center, Rotterdam, the Netherlands; 16 Heart Center Lucerne, Luzerner Kantonsspital, Lucerne, Switzerland; 17 Department of Cardiovascular Disease, German Heart Center Munich, Hamburg, Germany; 18 Department of Thoracic and Cardiovascular Surgery, Heart and Diabetes Center North Rhine-Westphalia, University Hospital Ruhr-University Bochum, Bad Oeynhausen, Germany; 19 Division of Cardiology, Pulmonology and Vascular Medicine, University Hospital Düsseldorf, Düsseldorf, Germany; 20 Asklepios Westklinikum Hamburg, Hamburg, Germany; 21 Cardiovascular Research Foundation, New York, NY USA; 22 Division of Cardiology, Rabin Medical Center, Petah Tikva, Israel; 23 Tel-Aviv University, Tel Aviv, Israel; 24 Department for General and Interventional Cardiology/Angiology, Heart and Diabetes Center North Rhine-Westphalia Bochum, University Hospital of the, University, Bad Oeynhausen, Germany

**Keywords:** Transcatheter aortic valve replacement, Sex, Gender, Prosthesis-patient mismatch, Outcomes

## Abstract

**Background:**

Women remain underrepresented in transcatheter aortic valve replacement (TAVR) trials, despite well-recognized anatomical differences that necessitate tailored procedural strategies. This study assessed sex-specific differences in baseline characteristics, procedural approaches, hemodynamic outcomes, and their impact on 5-year all-cause mortality.

**Methods:**

We analyzed data from 20,094 patients in the IMPPACT TAVR registry. Hemodynamic outcomes, including prosthesis-patient mismatch (PPM), were defined according to Valve Academic Research Consortium-3 criteria. Kaplan–Meier and Cox models assessed 5-year all-cause mortality, and logistic regression identified predictors of PPM.

**Results:**

Women comprised 49.1% of the cohort. They were older (81.4 vs. 80.2 years, *p* < 0.001), more symptomatic (NYHA ≥ III: 74.4% vs. 67.6%, *p* < 0.001), and more frequently received self-expanding valves (66.5% vs. 45.7%, *p* < 0.001). Post-TAVR indexed effective orifice areas were slightly larger in women (1.01 ± 0.28 vs. 0.99 ± 0.27cm^2^/m^2^, *p* < 0.001). Five-year mortality was lower in women (HR 0.81, 95% CI 0.76–0.86, *p* < 0.001). Severe PPM occurred less frequently in women (4.0% vs. 4.5%, *p* < 0.001) and was associated with increased mortality only in men (HR 1.32, 95% CI 1.10–1.59, *p* = 0.002). Adjustment for comorbidities nullified the association between PPM and mortality in both sexes.

**Conclusions:**

In this international TAVR cohort, women, despite being older, demonstrated better survival than men, with comparable postprocedural hemodynamic outcomes. Severe PPM was associated with increased mortality only in men, likely reflecting underlying comorbidities and low-flow states. After adjustment for confounders, PPM was no longer associated with survival in either sex. These findings underscore the need for strategies accounting for both procedural and patient-specific risks, beyond prosthesis hemodynamics.

**Graphical Abstract:**

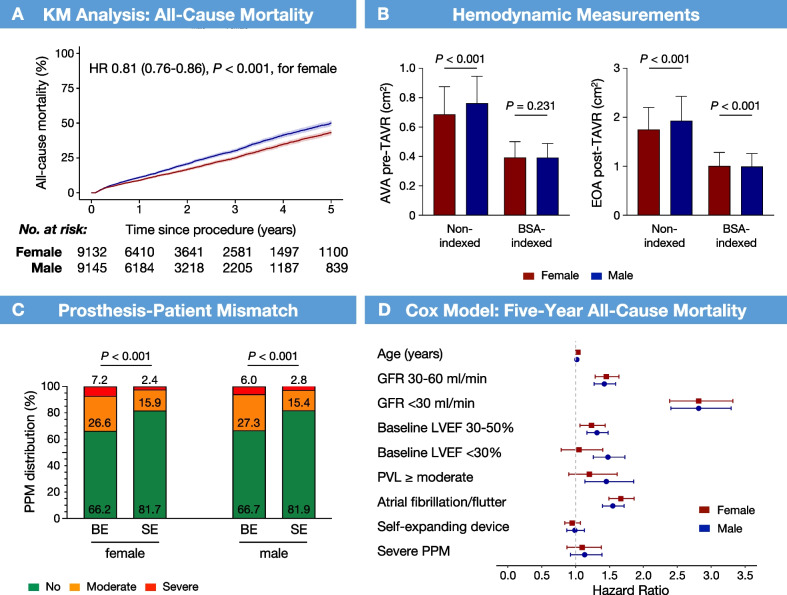

Sex-Specific Differences in TAVR, (**A**) Kaplan-Meier curves with 95% CI for all-cause mortality stratified by sex. (**B**) TTE-derived non-indexed and BSA-indexed pre-TAVR AVA and post-TAVR EOA stratified by sex. (**C**) Distribution of PPM severity stratified by device type and sex. (**D**) Forest plot showing results of the multivariable Cox proportional hazards model for 5-year all-cause mortality including a comparison of severe PPM against non-severe PPM (no and moderate) in men and women. HR with 95% CI are shown. Study center was included as a random effect in the model. Abbreviations: AVA: aortic valve area; BE: balloon-expandable; CI: confidence interval; EOA: effective orifice area; GFR: glomerular filtration rate; HR: hazard ratio; KM: Kaplan-Meier; LVEF: left ventricular ejection fraction; PPM: prosthesis-patient mismatch; PVL: paravalvular leak; SE: self-expanding

**Supplementary Information:**

The online version contains supplementary material available at 10.1007/s00392-025-02794-2.

## Introduction

Transcatheter aortic valve replacement (TAVR) has emerged as a significant advancement for the treatment of severe aortic stenosis (AS) [1, 2]. Notably, sex-specific differences have been observed: women typically present at an older age, with more symptomatic disease and smaller body size and annular dimensions than men [3, 4]. However, randomized data on procedural differences and outcomes remain limited, as women have been underrepresented in the large landmark trials comparing TAVR vs surgical aortic valve replacement (SAVR), especially in the most recent low-risk trials [1, 2]. This underrepresentation has prompted efforts to generate dedicated data focusing on women: the SMART trial showed superior hemodynamics with self-expanding (SE) valves in predominantly female patients with small annuli, and the RHEIA trial exclusively enrolled women and reported better 1-year clinical outcomes regarding mortality, stroke, and rehospitalization with TAVR compared to SAVR [5, 6]. While these studies offer important insights, their short follow-up and lack of detailed sex-stratified analyses leave the long-term relevance of sex-related anatomical and procedural differences—such as prosthesis–patient mismatch (PPM)—incompletely understood.

PPM occurs when the effective orifice area (EOA) of the implanted valve is too small relative to the patient's body surface area (BSA), potentially causing suboptimal hemodynamic performance [7]. Studies have reported discrepant findings regarding its clinical impact, possibly due to differences in study populations, valve types (e.g., balloon-expandable [BE] vs. SE devices), analytical approaches, or lack of sex-specific assessments [8–10]. Despite anatomical predisposition to PPM, women frequently achieve clinical outcomes equal or superior to men after TAVR [11, 12]. However, severe PPM is uncommon, and survival analyses alone may underestimate its potential sex-specific implications [13]. Moreover, procedural strategies, including valve selection, pre- and post-dilation, might have been applied differently among sexes to avoid the occurrence of PPM [14, 15].

Understanding the interplay of sex, anatomical characteristics, procedural strategies, and clinical outcomes is essential to optimize TAVR therapy. Therefore, this study aimed to evaluate sex-specific differences in pre- and postprocedural hemodynamics, and their relationship to long-term outcomes in a large, international TAVR cohort.

## Methods

### Study cohort

The IMPPACT TAVR (Impact of Measured or Predicted Prosthesis-pAtient mismatCh after Transcatheter Aortic Valve Replacement) registry retrospectively included patient-level data of 38,808 consecutive individuals receiving TAVR for severe native AS in 26 centers across Europe and Israel between 2006 and 2022 [10]. This study analyzed a subset of 20,094 patients with available echocardiographic data. Missing data were due to EOA measurements not performed or reported (10,287 patients) and echocardiographic data not submitted by the participating centers (8,427 patients). Valve-in-valve cases were excluded. The decision to perform TAVR was made by the heart team at each center. The cohort included patients who received valves from Edwards Lifesciences (SAPIEN XT, SAPIEN 3, SAPIEN 3 Ultra), Medtronic (CoreValve, Evolut R, Evolut PRO, Evolut PRO +), or Symetis/Boston Scientific (ACURATE neo, ACURATE neo2). Procedural specifics, including valve choice, were determined by each center. The primary study outcome was 5-year all-cause mortality. Sex was recorded as biological sex; no information on gender identity was available. The study followed the Declaration of Helsinki, was approved by the local ethics board, and registered in the German Clinical Trials Register (DRKS00035691). The research reported in this paper adhered to the STROBE guidelines.

### Hemodynamic assessment

Hemodynamic parameters were derived from pre-procedural (within 3 months) and discharge transthoracic echocardiograms (TTEs). All echocardiographic outcomes were site-reported. Parameters included mean transvalvular pressure gradient (dPmean), change in dPmean, non-indexed and BSA-indexed EOA/EOAi, and the presence and severity of paravalvular leak (PVL). BSA was determined using the DuBois formula. PPM was categorized based on the Valve Academic Research Consortium-3 consensus: severe for EOAi ≤ 0.65 cm^2^/m^2^, moderate for EOAi ≤ 0.85 cm^2^/m^2^ (adjusted for body mass index (BMI) ≥ 30 kg/m^2^: severe ≤ 0.55 cm^2^/m^2^, moderate ≤ 0.7 cm^2^/m^2^) [16]. EOA calculations were performed using the continuity equation. Left ventricular outflow tract (LVOT) was measured during mid-systole in the parasternal long-axis view from the outer-edge to the outer-edge of the stented valve at the ventricular end. LVOT flow was measured at the same level with pulsed-wave Doppler [17]. All PPM analyses used BMI-adjusted EOA values.

### Statistical analysis

Categorical variables were analyzed using chi-square statistics. For continuous variables, normality was assessed using the Kolmogorov–Smirnov test. Student’s t-test or ANOVA were applied to normally distributed data, while Mann–Whitney U or Kruskal–Wallis tests were used for non-normal data. Five-year all-cause mortality was assessed using Kaplan–Meier estimates and log-rank tests. Additionally, multivariable Cox proportional hazard models were used, including key variables that have been consistently linked with post-TAVR mortality (age, left ventricular ejection fraction [LVEF], glomerular filtration rate [GFR], atrial fibrillation/flutter [AF], and paravalvular leak [PVL]) as well as variables of procedural interest (device type and PPM) [18–20]. Severe PPM was compared to non-severe (no and moderate) in indicated multivariable models. Operating center was included as a random effect. Hazard ratios (HR) and 95% confidence intervals (CI) were calculated. The proportional hazards assumption was verified through graphical methods and goodness of fit tests (all *P* values statistically not significant). Mortality analysis was limited to patients with follow-up beyond 30 days to reduce procedural impact. To specifically address shorter-term outcomes, 30-day mortality was calculated as an observed proportion and compared using chi-square statistics. To further explore short-term associations between PPM severity and 30-day mortality, sex-specific logistic regression models were used; results are presented as odds ratios (OR) with 95% CI. Predictors of severe PPM were identified via multivariable logistic regression. Covariates for this analysis were selected a priori based on clinical relevance and their potential influence on EOA or flow dynamics, including BMI, annulus area, LVEF, AF, and device type. Aortic annulus area was divided by quartiles. Statistical significance was assumed at *p* < 0.05. All analyses were performed using SPSS v28.0 (IBM, Armonk, NY, USA), GraphPad Prism v10.1 (Dotmatics, Boston, MA, USA), and R v4.3.0 (R Core Team, Vienna, Austria, 2023).

## Results

### Baseline characteristics

**Fig. 1:**
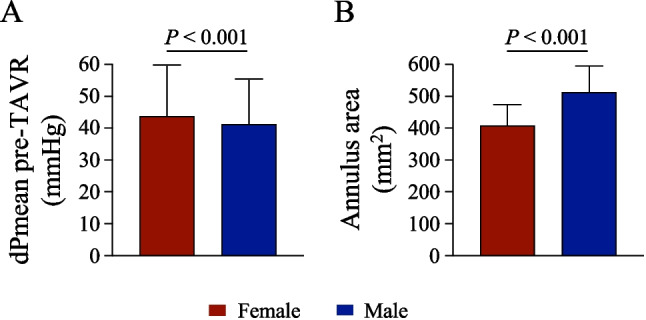
Pre-TAVR Cardiovascular Measurements, (**A**) Pre-TAVR mean transvalvular pressure gradients were higher in female patients. (**B**) CT-derived annulus area was larger in males. Mean and standard deviation are shown. Abbreviations: CT: computed tomography; dPmean: mean transvalvular pressure gradient

Of the 20,094 patients, 49.1% were women. The mean age was 80.8 ± 6.2 years and the mean Society of Thoracic Surgeons (STS) Score was 5.2 ± 4.4% (Table 1). Women were older (81.4 ± 5.9 vs. 80.2 ± 6.4 years, *p* < 0.001) and had a smaller BSA (1.75 ± 0.19 vs. 1.96 ± 0.18m^2^, *p* < 0.001), yet with a comparable BMI (27.7 ± 5.8 vs. 27.5 ± 4.6, *p* = 0.886). Furthermore, women exhibited a higher STS score (5.9 ± 4.6 vs. 4.6 ± 4.2, *p* < 0.001), which includes female sex as a risk factor. More females were classified as New York Heart Association (NYHA) functional class ≥ III (74.4 vs. 67.6%, *p* < 0.001). Males had a higher burden of comorbidities, including chronic obstructive pulmonary disease, coronary artery disease (CAD), diabetes, hyperlipidemia, AF, and prior stroke. They also more frequently had a history of pacemaker implantation and prior cardiac surgery. While baseline native aortic valve area (AVA) was smaller in women (0.69 ± 0.19 vs. 0.76 ± 0.18cm^2^, *p* < 0.001), there were no differences in AVA when indexed to BSA (0.39 ± 0.11 vs. 0.39 ± 0.10cm^2^/m^2^, *p* = 0.231) (Graphic Abstract). However, pre-TAVR dPmean (43.8 ± 16.0 vs. 41.2 ± 14.2mmHg, *p* < 0.001) and LVEF (57.3 ± 11.0 vs. 52.6 ± 13.0%, *p* < 0.001) were slightly higher in women (Fig. 1A). Additional baseline characteristics are presented in Table 1.

**Table 1:** Baseline characteristics

Parameter	All	Female	Male	*P* value
N	20,094	9860 (49%)	10,234 (51%)	
*Age – years*	80.8 ± 6.2	81.4 ± 5.9	80.2 ± 6.4	< 0.001
*BMI – kg/m*^*2*^	27.6 ± 5.2	27.7 ± 5.8	27.5 ± 4.6	0.886
*BSA – m*^*2*^	1.86 ± 0.21	1.75 ± 0.19	1.96 ± 0.18	< 0.001
*STS score – %*	5.2 ± 4.4	5.9 ± 4.6	4.6 ± 4.2	< 0.001
*STS category*				< 0.001
* Low-risk (*< *4%)*	8034 (40.0%)	3304 (33.5%)	4730 (46.2%)	
*Intermediate-risk (4–8%)*	5316 (26.5%)	3000 (30.4%)	2316 (22.6%)	
*High-risk (*> *8%)*	6744 (33.6%)	3556 (36.1%)	3188 (31.2%)	
*Pre-TAVR dPmean – mmHg*	42.5 ± 15.2	43.8 ± 16.0	41.2 ± 14.2	< 0.001
*Low-gradient AS*				< 0.001
*dPmean 30–40 mmHg*	4439 (23.4%)	2090 (22.5%)	2349 (24.4%)	
*dPmean* < *30 mmHg*	3720 (19.6%)	1701 (18.3%)	2019 (21.0%)	
*Pre-TAVR LVEF – %*	54.9 ± 12.3	57.3 ± 11.0	52.6 ± 13.0	< 0.001
*NYHA class* ≥ *III*	14,214 (71.0%)	7318 (74.4%)	6896 (67.6%)	< 0.001
*Comorbidities*				
*GFR – ml/min*	62.1 ± 21.5	60.1 ± 21.2	64.0 ± 21.6	< 0.001
*Hypertension*	16,694 (90.1%)	8230 (90.6%)	8464 (89.7%)	0.031
*COPD*	2017 (14.5%)	890 (13.3%)	1127 (15.7%)	< 0.001
*CAD*	11,493 (57.2%)	4661 (47.3%)	6832 (66.8%)	< 0.001
*Diabetes*	6612 (33.3%)	3099 (31.7%)	3513 (34.8%)	< 0.001
*Hyperlipidemia*	6430 (60.9%)	2935 (57.0%)	3495 (64.6%)	< 0.001
*Atrial fibrillation/flutter*	7402 (36.9%)	3399 (34.5%)	4003 (39.2%)	< 0.001
*Prior stroke/TIA*	4038 (20.6%)	1885 (19.6%)	2153 (21.6%)	< 0.001
*Prior cardiac surgery*	2018 (10.0%)	577 (5.9%)	1441 (14.1%)	< 0.001
*Prior pacemaker*	2311 (11.5%)	885 (9.0%)	1426 (13.9%)	< 0.001

Baseline characteristics of the entire study population and stratified by sex. Continuous values are presented as mean ± SD, categorical values as n (%)

BMI: body mass index; BSA: body surface area; CAD: coronary artery disease; COPD: chronic obstructive pulmonary disease; dPmean; mean transvalvular pressure gradient; GFR: glomerular filtration rate; LVEF: left ventricular ejection fraction; PVL: paravalvular leak; SD: standard deviation; STS: Society of Thoracic Surgeons; TIA: transient ischemic attack

### Procedural and hemodynamic outcomes

**Fig. 2:**
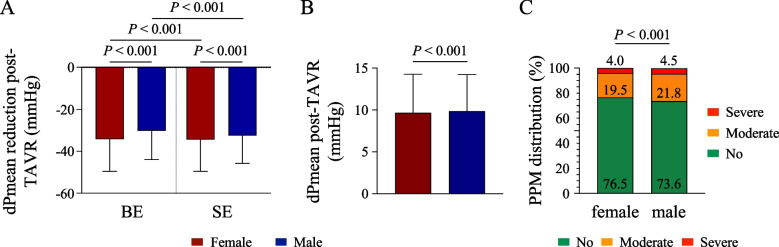
Post-TAVR Cardiovascular Measurements and PPM distribution, (**A**) Females exhibited a greater decline in dPmean post-TAVR than males in both BE and SE devices. (**B**) Absolute dPmean was higher in males, yet clinically comparable between sexes. (**C**) Moderate and severe PPM were less frequently observed in female compared to male patients, Mean and standard deviation are shown. Abbreviations: BE: balloon-expandable; dPmean: mean transvalvular pressure gradient; PPM: prosthesis-patient mismatch; SE: self-expanding

Computed tomography (CT)-derived annulus area was smaller in women (409 ± 65 vs. 513 ± 82mm^2^, *p* < 0.001) (Fig. 1B). They received more SE devices than males (66.5 vs. 45.7%, *p* < 0.001) (Table 2). Details regarding the number and sizes of implanted valves are shown in Supplementary Table 1A and B. Females underwent a higher proportion of pre- (60.3 vs. 52.1%) and post-dilation (24.4 vs. 21.7%, *p* < 0.001 for both) than men, yet with comparable rates of at least moderate paravalvular leak (PVL) (3.6 vs. 3.4%, *p* = 0.409). Post-TAVR, females showed a greater decline in dPmean in both BE (−32.5 ± 15.0 vs. −30.4 ± 13.4 mmHg, *p* < 0.001) and SE devices (−34.8 ± 14.6 vs. −32.8 ± 13.1 mmHg, *p* < 0.001) (Fig. 2A). However, post-TAVR dPmean remained slightly higher in females in BE (12.0 ± 4.9 vs. 11.3 ± 4.3 mmHg, *p* < 0.001) and SE devices (8.5 ± 4.0 vs. 8.2 ± 3.8 mmHg, *p* < 0.001) (Supplementary Fig. 1). Due to the more frequent use of SE valves, overall post-TAVR dPmean was slightly lower in women (9.7 ± 4.6 vs. 9.9 ± 4.4mmHg, *p* < 0.001) (Fig. 2B). Non-indexed TAVR EOA was larger in men (1.75 ± 0.45 vs. 1.93 ± 0.50cm^2^, *p* < 0.001), yet clinically comparable when indexed to BSA (1.01 ± 0.28 in women vs. 0.99 ± 0.27cm^2^ in men, *p* < 0.001) (Graphic Abstract).

**Table 2:** Procedural characteristics and early outcomes

Parameter	All	Female	Male	*P* value
N	20,094	9860 (49.1%)	10,234 (50.9%)	
*Annulus area – mm*^*2*^	462 ± 91	409 ± 64	513 ± 82	< 0.001
*Annulus perimeter – mm*	76 ± 7	72 ± 5	80 ± 7	< 0.001
*Self-expanding device*	11,236 (55.9%)	6558 (66.5%)	4678 (45.7%)	< 0.001
*Pre-dilation*	11,093 (56.2%)	5842 (60.3%)	5251 (52.1%)	< 0.001
*Post-dilation*	4562 (23.0%)	2369 (24.4%)	2193 (21.7%)	< 0.001
*Transfemoral access*	19,131 (95.2%)	9421 (95.6%)	9710 (94.9%)	0.026
*Post-TAVR dPmax – mmHg*	17.8 ± 8.0	17.6 ± 8.2	18.1 ± 7.8	< 0.001
*Post-TAVR dPmean – mmHg*	9.8 ± 4.5	9.7 ± 4.6	9.9 ± 4.4	< 0.001
*Post-TAVR EOA – cm*^*2*^	1.84 ± 0.48	1.75 ± 0.45	1.93 ± 0.50	< 0.001
*Post-TAVR BSA-indexed EOA – cm*^*2*^*/m*^*2*^	0.99 ± 0.28	1.01 ± 0.28	0.99 ± 0.27	< 0.001
*Post-TAVR PVL* ≥ *moderate*	608 (3.5%)	306 (3.6%)	302 (3.4%)	0.409
*30-day mortality*	277 (1.4%)	131 (1.3%)	146 (1.4%)	0.548

Procedural characteristics and early outcomes of the entire study population and stratified by sex. Continuous values are presented as mean ± SD, categorical values as n (%)

BSA: body surface area; dPmean: mean transvalvular pressure gradient; EOA: effective orifice area; PVL: paravalvular leak

Although early mortality at 30 days did not differ by sex (1.3% in women vs. 1.4% in men, *p* = 0.548), Kaplan–Meier analysis demonstrated lower 5-year all-cause mortality in women (HR 0.81, 95% CI 0.76–0.86, *p* < 0.001), with an estimated mortality rate of 43.2% (95% CI 41.4–44.9%) vs. 50.1% (95% CI 48.1–51.9%) in men (Graphic Abstract). In multivariable Cox analysis female sex remained independently associated with lower 5-year all-cause mortality (HR 0.78, 95% CI 0.70–0.83, *p* < 0.001). Other independent predictors of higher mortality included older age, lower GFR, reduced LVEF, presence of AF, and ≥ moderate PVL. Notably, severe PPM was not significantly associated with 5-year mortality (HR 1.11, 95% CI 0.95–1.29, *p* = 0.193) (Table 3).

**Table 3:** Multivariable cox proportional hazards analysis of 5-year all-cause mortality

Parameter	HR (95% CI)	*P*
*Age (years)*	1.03 (1.02–1.03)	** < 0.001**
*GFR (ref:* > *60 ml/min)*		
*30–60 ml/min*	1.44 (1.33–1.56)	** < 0.001**
< *30 ml/min*	2.82 (2.52–3.16)	** < 0.001**
*Baseline LVEF (ref:* > *50%)*		
*30–50%*	1.28 (1.17–1.40)	** < 0.001**
< *30%*	1.34 (1.17–1.53)	** < 0.001**
*PVL* ≥ *moderate*	1.34 (1.11–1.62)	**0.002**
*Atrial fibrillation/flutter*	1.60 (1.49–1.73)	** < 0.001**
*Self-expanding device*	0.99 (0.91–1.07)	0.717
*Severe PPM*	1.11 (0.95–1.29)	0.193
*Female sex*	**0.78 (0.70–0.83)**	** < 0.001**

Cox proportional hazards analysis of 5-year all-cause mortality. Models were adjusted for clinically relevant variables (age, GFR, LVEF, PVL, Atrial fibrillation/flutter, device type and PPM). Study center was included as a random effect. HR are reported with 95% CI

CI: confidence interval; GFR: glomerular filtration rate; HR: hazard ratio; LVEF: left ventricular ejection fraction; PPM: prosthesis-patient mismatch; PVL: paravalvular leak

### Prevalence and impact of PPM post-TAVR

Moderate and severe PPM occurred less frequently in women than in men (19.5% vs. 21.8% and 4.0% vs. 4.5%, *p* < 0.001 for both) (Fig. 2C). Irrespective of sex, PPM was more frequent in BE than in SE devices (Graphic Abstract).

**Fig. 3:**
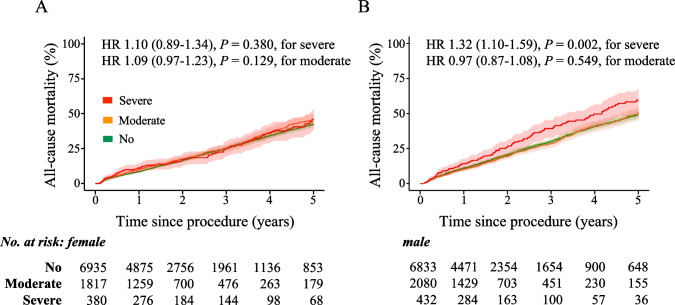
Sex-specific 5-Year All-Cause Mortality Stratified by PPM Severity, Kaplan–Meier curves with 95% CI for all-cause mortality in the (**A**) female and (**B**) male patient cohort stratified by the presence and severity of PPM. While PPM had no impact on mortality in female patients, it adversely affected survival in male patients. Abbreviations: CI: confidence interval; HR: hazard ratio; PPM: prosthesis-patient mismatch

In Kaplan–Meier analysis neither moderate (HR 1.09, 95% CI 0.97–1.23, *p* = 0.129) nor severe PPM (HR 1.10, 95% CI 0.89–1.34, *p* = 0.380) were associated with increased mortality in women, with an estimated mortality rate at 5 years of 42.3% (95% CI 40.3–44.4%) in the absence of PPM, 45.7% (95% CI 41.3–49.8%) with moderate PPM, and 45.8% (95% CI 37.9–52.7%) with severe PPM. In contrast, severe PPM was significantly associated with increased mortality in men (HR 1.32, 95% CI 1.10–1.59, *p* = 0.002), while moderate PPM had no impact on mortality (HR 0.97, 95% CI 0.87–1.08, *p* = 0.549). The estimated mortality rate at 5 years in men was 49.4% (95% CI 47.1–51.5%) in the absence of PPM, 50.3% (95% CI 45.7–54.5%) with moderate PPM, and 59.4% (95% CI 50.5–66.8%) with severe PPM, respectively (Fig. 3).

In multivariable Cox analysis, severe PPM, as compared to non-severe PPM, did not significantly affect 5-year all-cause mortality in women (HR 1.09, 95% CI 0.94–1.46, *p* = 0.456) or men (HR 1.13, 95% CI 0.87–1.38, *p* = 0.231) (Graphic Abstract).

With respect to short-term outcomes, no significant association between PPM severity and 30-day mortality was observed in either sex (severe vs. no PPM: women, OR 0.52, 95% CI 0.13–1.38, *p* = 0.261; men, OR 1.18, 95% CI 0.53–2.29, *p* = 0.651). In exploratory analyses including only patients with preserved LVEF (≥ 50%), similar results were obtained, supporting the robustness of our findings (data not shown).

### Factors associated with severe PPM

Baseline characteristics of the male and female patient cohort stratified by presence and severity of PPM are shown in Supplementary Table 2 and 3. In multivariable binary logistic regression analysis, SE device use was protective against severe PPM in both sexes (women: OR 0.32, 95% CI 0.24–0.41, *p* < 0.001; men: OR 0.40, 95% CI 0.30–0.52, *p* < 0.001). Predictors of severe PPM included LVEF 30–50% (women: OR 1.60, 95% CI 1.14–2.21, *p* = 0.005; men: OR 1.71, 95% CI 1.31–2.22, *p* < 0.001), LVEF < 30% (women: OR 3.31, 95% CI 1.92–5.42, *p* < 0.001; men: OR 1.95, 95% CI 1.34–2.80, *p* < 0.001), AF (women: OR 1.56, 95% CI 1.18–2.06, *p* = 0.002; men: OR 1.77, 95% CI 1.39–2.25, *p* < 0.001), and annulus area < 400 mm^2^ (women: OR 2.49, 95% CI 1.36–5.04, *p* = 0.006; men: OR 1.81, 95% CI 1.07–2.92, *p* = 0.020) (Table 4).

**Table 4:** Sex-specific predictors of severe prosthesis-patient mismatch

Parameter	OR (95% CI)	*P*	OR (95% CI)	*P*
	Female		Male	
*BMI*	1.02 (1.00–1.04)	0.081	1.01 (0.98–1.03)	0.537
*Atrial fibrillation/flutter*	1.56 (1.18–2.06)	0.002	1.77 (1.39–2.25)	< 0.001
*Self-expanding device*	0.32 (0.24–0.41)	< 0.001	0.40 (0.30–0.52)	< 0.001
*Baseline LVEF (ref:* > *50%)*				
*30–50%*	1.60 (1.14–2.21)	0.005	1.71 (1.31–2.22)	< 0.001
< *30%*	3.31 (1.92–5.42)	< 0.001	1.95 (1.34–2.80)	< 0.001
*Annulus area (ref:* > *520cm*^*2*^*)*				
*450-520cm*^*2*^	1.21 (0.61–2.56)	0.599	1.11 (0.84–1.47)	0.467
*400-449cm*^*2*^	1.88 (1.00–3.86)	0.064	1.32 (0.88–1.94)	0.165
< *400cm*^*2*^	2.49 (1.36–5.04)	0.006	1.81 (1.07–2.92)	0.020

Multivariable binary logistic regression analysis of predictors for the occurrence of severe prosthesis-patient mismatch, separated by sex. OR are reported with 95% CI

Abbreviations: CI: confidence interval; OR: odds ratio; LVEF: left ventricular ejection fraction

Women with severe PPM were older (80.2 ± 7.0 vs. 78.9 ± 7.3 years, p = 0.006), had higher pre-TAVR LVEF (54.4 ± 13.1 vs. 48.3 ± 14.0%, *p* < 0.001), smaller annuli (395 ± 70 vs. 519 ± 79mm^2^, *p* < 0.001) and received more SE valves (40.0% vs. 28.0%, *p* < 0.001) than men. They also had lower rates of CAD (49.6% vs. 65.5%, *p* < 0.001) and AF (39.7% vs. 52.5%, *p* < 0.001) (Supplementary Table 4).

## Discussion

This international registry study provides—to the best of our knowledge—the largest and most comprehensive sex-stratified analysis based on patient-level data, linking detailed hemodynamic parameters—including valve type, EOA, and PPM—with long-term outcomes after TAVR. By integrating anatomical, procedural, and clinical endpoints, our study reveals new insights into sex-specific disparities in TAVR. Key findings include:

- Despite being older and more symptomatic, women exhibited a survival advantage during 5-year follow-up.
- Although women had smaller aortic annuli and higher pre-TAVR pressure gradients, post-TAVR hemodynamics were similar across sexes.
- Women were more likely to receive SE valves, had lower rates of PPM, and a similar incidence of at least moderate PVL.
- Severe PPM was associated with higher mortality in men, but not in women; however, this association was mitigated after adjustment for baseline comorbidities.

### Baseline characteristics, procedural approaches and hemodynamic outcomes

Consistent with prior studies, women presented at an older age with more advanced symptoms but had fewer comorbidities such as CAD and AF [3, 15]. Delayed referral or under recognition of symptoms may contribute to the higher NYHA class at presentation [4]. Recent surveys revealed that treatment is often withheld in women due to age, frailty, patient refusal, or decrease of symptoms after conservative treatment, highlighting potential disparities in care delivery [4].

Despite significant differences in symptoms, pre-TAVR indexed AVA was comparable between sexes, suggesting that current guideline thresholds, which do not account for anatomical differences, may inadequately identify women who would benefit from intervention [21]. This underscores the need for further research to identify sex-specific thresholds, ensuring appropriate treatment irrespective of sex.

Regarding procedural approaches, women were more likely to receive SE valves, which more often require pre- and post-dilation [22]. This pattern mirrors the SMART trial, where SE valves yielded larger indexed EOAs and superior hemodynamics in a predominantly female, small-annulus cohort, although the clinical implications for long-term survival remain to be elucidated [5]. These tailored procedural strategies successfully addressed anatomical challenges in women, leading to comparable EOAs post-TAVR and demonstrating the value of individualized procedural planning. Additionally, women had greater declines of pressure gradients in both BE and SE devices, which may be attributed to their higher baseline gradients and/or reflect sex-specific hemodynamic responses to TAVR [23].

### Sex-specific survival after TAVR

Though being older and more symptomatic, women demonstrated better survival at 5-year follow-up, likely influenced by their overall lower comorbidity burden [11, 24]. This favorable survival after TAVR contrasts with some reports suggesting lower long-term survival in women with severe AS, possibly due to lower rates of interventions or treatment at more advanced disease stages [3]. Our findings, however, indicate that when interventions like TAVR are performed, women can experience excellent outcomes, highlighting the benefits of timely and appropriate treatment. This observation aligns with findings from the RHEIA trial, which demonstrated early clinical benefits of TAVR over SAVR in female patients, reinforcing the need to ensure equitable access to transcatheter therapies for women [6]. This perspective is further supported by the recent ESC/EACTS guidelines, which now recommend TAVR in selected asymptomatic patients (Class IIa), emphasizing the importance of timely intervention in aortic stenosis [25].

Beyond their overall lower comorbidity burden, additional factors may contribute to the observed survival advantage in women, despite similar post-TAVR hemodynamics. Sex-specific differences in myocardial remodeling might play a crucial role. Some studies suggest that women may exhibit more favorable left ventricular remodeling in response to the pressure overload caused by AS, characterized by less fibrosis and collagen deposition [26]. This could allow for faster and more effective reversal of cardiac remodeling after TAVR. Better preserved microvascular function may further support their observed survival advantage [27, 28].

### Incidence, impact and predictors of PPM

Our study identified two main mechanisms contributing to severe PPM: first, anatomical and procedural factors; and second, indicators of low-flow physiology. Anatomically, smaller annulus dimensions inherently limit the size of the prosthetic valve that can be implanted, thereby increasing the risk of a smaller EOA. Procedurally, SE valves may reduce the risk of PPM due to their supra-annular design and have been shown to outperform BE devices in terms of hemodynamic performance, particularly in small annuli [5]. In addition, low-flow conditions may lead to underestimation of EOA due to incomplete valve opening, resulting in overclassification of PPM and the appearance of pseudo-severe PPM. This phenomenon poses a diagnostic challenge, as many conventional echocardiographic indices are highly flow dependent and may not accurately reflect true prosthetic valve performance under reduced stroke volume conditions. Such low-flow states may, however, be associated with an increased mortality risk primarily driven by the underlying comorbidities that cause reduced stroke volume (e.g., heart failure with reduced or preserved ejection fraction, significant mitral regurgitation, or atrial fibrillation). This underscores the need for novel flow-independent approaches to assess valve performance and its impact on clinical outcomes [18, 20, 29].

Women had lower rates of PPM, indicating that procedural adaptations like SE valve use effectively resulted in larger EOAs despite anatomical constraints. Additionally, better LVEF and lower AF prevalence may contribute to lower PPM rates in women, while in men worse LVEF and a higher AF burden may lead to overestimation of true PPM frequency and severity. While the same predictors applied to both sexes, baseline differences were notable. Women with increasing PPM severity also had worse LVEF and higher AF frequency, but their LVEF remained within the normal range, and AF prevalence was significantly lower than in men (Supplementary Table 4). Women may therefore be more frequently classified as having severe PPM due to true mismatch rather than pseudo-severe PPM.

Importantly, severe PPM was associated with increased mortality only in men. However, this association was nullified after adjustment for comorbidities in a multivariable model. This suggests that PPM serves as a surrogate marker of adverse risk profiles rather than a direct causal factor. This highlights the importance of competing risks of death in TAVR populations and underscores the need for closer surveillance and individualized management in this high-risk subgroup [18, 20]. Moreover, even in cases of true severe PPM, the hemodynamic relief from resolving severe AS may be sufficient to mitigate the adverse impact of mismatch on survival. PPM may nevertheless carry greater prognostic weight in selected subgroups—such as patients with markedly reduced LVEF or persistent low-flow states— where elevated residual afterload and impaired ventricular function may synergistically worsen outcomes [30].

### Limitations

Several limitations should be acknowledged: Potential confounders, inaccuracies, incomplete data, and selection bias inherent to registry-based studies may influence the results. While the study reflects real-world practice, echocardiographic measurements lack core lab adjudication, which could affect the consistency of findings. Although adjustments were made for multiple variables, unmeasured factors could still impact outcomes. Importantly, differentiation between true and pseudo-severe PPM was not feasible in this registry and may have influenced the observed associations between PPM and outcomes. Finally, the generalizability of our findings may be limited to similar patient populations and specific TAVR devices.

## Conclusion

This study underscores the importance of sex-specific considerations in TAVR and complements recent randomized trials such as SMART and RHEIA by providing long-term, sex-stratified outcome data. The survival benefit in women, despite being older and more symptomatic, highlights the potential value of timely intervention, which may be delayed under current guideline thresholds for aortic stenosis that overlook anatomical differences. In men, a diagnosis of severe PPM identified a high-risk subgroup requiring close monitoring; however, this risk was driven by underlying comorbidities rather than true anatomical mismatch, as PPM was not independently associated with mortality after adjustment in either sex. This challenges the focus on procedural adaptations solely aimed at avoiding PPM. These results support a holistic, patient-specific approach to TAVR that integrates clinical and anatomical risk factors to optimize outcomes in both sexes. Future research should evaluate sex-specific thresholds for aortic stenosis as well as flow-independent assessments of valve performance and their impact on clinical outcomes.

## Supplementary Information

Below is the link to the electronic supplementary material.

Supplementary file1 (DOCX 89 KB)

## Data Availability

Data are available from the corresponding author upon reasonable request.

## Abbreviations

AVA: Aortic Valve Area (native, pre-TAVR)
BSA: Body Surface Area
BE: Balloon-Expandable
dPmean: Mean Transvalvular Pressure Gradient
EOA: Measured Effective Orifice Area of the Implanted Valve (post-TAVR)
PPM: Measured Prosthesis-Patient Mismatch
SE: Self-Expanding
TAVR: Transcatheter Aortic Valve Replacement
TTE: Transthoracic Echocardiography

## Declarations

Dr Bleiziffer received speaker honoraria from Abbott, Boston Scientific, Edwards Lifesciences, and Medtronic. Dr Bunc served as proctor for TAVR procedures with Edwards Lifesciences, Medtronic, Meril, and Abbott; is a member of the advisory boards for Medtronic and Edwards Lifesciences. Dr Van Mieghem received institutional research grants from Abbott Vascular, Boston Scientific, Medtronic, Teleflex, PulseCath BV and advisory fees from Abbott Vascular, Boston Scientific, Medtronic, Anteris, JenaValve, Siemens, Materialise, Pie Medical, Luma Vision, Daiichi Sankyo, Bolt Medical. Dr Abdel-Wahab’s institution has received speaker’s honoraria and/or consultancy fees from Boston Scientific, and Medtronic. Dr Kim has received proctor and personal fees from Abbott, Boston Scientific, Meril Life Sciences, and Shockwave Med; is a member of the advisory boards for Abbott, Boston Scientific, and HID Imaging; and Dr Kim’s institution has received fees from Boston Scientific. Dr Nombela-Franco has served as proctor for Abbott and Edwards Lifesciences. Dr Rudolph received lecture fees from Abbott, SIS Medical and Translumina. Dr Schmidt is proctor for Medtronic and Boston and received speaker honoraria and travel expenses from Medtronic, Boston, Meril and Edwards Lifesciences. Dr Toggweiler has received honoraria from Medtronic, Boston Scientific, Biosensors, Hi-D Imaging, Abbott Vascular, Medira, Shockwave, Teleflex, atHeart Medical, Cardiac Dimensions, Polares Medical, Amarin, Sanofi, AstraZeneca, ReCor Medical, Daiichi Sankyo; has received institutional research grants from Edwards Lifesciences, Abbott Vascular, Boston Scientific, Fumedica, Novartis, Boehringer Ingelheim, Polares Medical, and holds equity in Hi-D Imaging. Dr Veulemans has received speaker and/or study honoraria from Edwards Lifesciences, Medtronic, and Boston Scientific. Dr Wienemann has received travel grants from JenaValve. Dr Witberg has received consultancy fees from Medtronic. Dr Zeus received speaker and/or study honoraria from Edwards Lifesciences, Medtronic, and Boston Scientific.

All other authors have reported that they have no relationships relevant to the contents of this paper to disclose.
