# Supplementary material for: Sex differences in hemodynamics and outcomes after transcatheter aortic valve replacement
Source: Clin Res Cardiol. 2025 Nov 20;115(7):1207–17. doi: 10.1007/s00392-025-02794-2 (PMC13249733; doi:10.1007/s00392-025-02794-2)
Supplement: Supplementary file 1 — Supplementary file1 (DOCX 89 KB) [file 392_2025_2794_MOESM1_ESM.docx]

**Supplementary Table 1A: Number of Implanted Balloon-Expandable Devices**

| **BE Device** | **Size (mm)** | **Female n (% of BE)** | **Male n (% of BE)** |
| --- | --- | --- | --- |
| Sapien XT | Total | 686 (20.8 %) | 528 (9.5 %) |
|  | 23 | 249 (7.5 %) | 71 (1.3 %) |
|  | 26 | 369 (11.2 %) | 278 (5.0 %) |
|  | 29 | 68 (2.1 %) | 179 (3.2 %) |
| Sapien 3 | Total | 2010 (60.8 %) | 4047 (72.9 %) |
|  | 20 | 28 (0.8 %) | 2 (0.0 %) |
|  | 23 | 1082 (32.8 %) | 241 (4.3 %) |
|  | 26 | 704 (21.3 %) | 1490 (26.8 %) |
|  | 29 | 196 (5.9 %) | 2314 (41.7 %) |
| Sapien 3 Ultra | Total | 606 (18.4 %) | 981 (17.7 %) |
|  | 20 | 22 (0.7 %) | 1 (0.0 %) |
|  | 23 | 344 (10.4 %) | 80 (1.4 %) |
|  | 26 | 234 (7.1 %) | 781 (14.1 %) |
|  | 29 | 6 (0.2 %) | 119 (2.1 %) |
| **Total** |  | **3302 (100%)** | **5556 (100%)** |

Number of implanted balloon-expandable (BE) valves by device type and size, stratified by sex.

**Supplementary Table 1B: Number of Implanted Self-Expanding Devices**

| **SE Device** | **Size (mm)** | **Female n (% of SE)** | **Male n (%of SE)** |
| --- | --- | --- | --- |
| CoreValve | Total | 885 (13.5 %) | 733 (15.7 %) |
|  | 23 | 56 (0.9 %) | 15 (0.3 %) |
|  | 26 | 452 (6.9 %) | 73 (1.6 %) |
|  | 29 | 354 (5.4 %) | 506 (10.8 %) |
|  | 31 | 23 (0.4 %) | 139 (3.0 %) |
| Evolut R | Total | 1895 (28.9 %) | 1502 (32.1 %) |
|  | 23 | 132 (2.0 %) | 16 (0.3 %) |
|  | 26 | 796 (12.1 %) | 112 (2.4 %) |
|  | 29 | 893 (13.6 %) | 672 (14.4 %) |
|  | 34 | 74 (1.1 %) | 702 (15.0 %) |
| Evolut PRO | Total | 535 (8.2 %) | 427 (9.1 %) |
|  | 23 | 19 (0.3 %) | 0 (0.0 %) |
|  | 26 | 226 (3.4 %) | 41 (0.9 %) |
|  | 29 | 290 (4.4 %) | 386 (8.3 %) |
| Evolut PRO+ | Total | 213 (3.2 %) | 272 (5.8 %) |
|  | 23 | 5 (0.1 %) | 0 (0.0 %) |
|  | 26 | 82 (1.3 %) | 12 (0.3 %) |
|  | 29 | 114 (1.7 %) | 156 (3.3 %) |
|  | 34 | 12 (0.2 %) | 104 (2.2 %) |
| ACURATE neo | Total | 1941 (29.6 %) | 1098 (23.5 %) |
|  | 23 | 667 (10.2 %) | 83 (1.8 %) |
|  | 25 | 935 (14.3 %) | 377 (8.1 %) |
|  | 27 | 339 (5.2 %) | 638 (13.6 %) |
| ACURATE neo2 | Total | 1089 (16.6 %) | 646 (13.8 %) |
|  | 23 | 414 (6.3 %) | 18 (0.4 %) |
|  | 25 | 498 (7.6 %) | 225 (4.8 %) |
|  | 27 | 177 (2.7 %) | 403 (8.6 %) |
| **Total** |  | **6558 (100%)** | **4678 (100%)** |

Number of implanted self-expanding (SE) valves by device type and size, stratified by sex.

**Supplementary Table 2: Characteristics of the Female Patient Cohort**

| **Parameter** | **PPM:**  **no** | **PPM:**  **moderate** | **PPM:**  **severe** | ***P* value** |
| --- | --- | --- | --- | --- |
| *N* | 7542 | 1923 | 395 |  |
| *Age - years* | 81.6±5.8 | 81.0±6.0 | 80.2±7.0 | **<0.001** |
| *BMI - kg/m^2^* | 27.6±5.8 | 27.7±5.6 | 27.7±5.9 | 0.544 |
| *BSA - m^2^* | 1.74±0.18 | 1.79±0.18 | 1.80±0.20 | **<0.001** |
| *STS Score - %* | 5.9±4.6 | 5.7±4.3 | 6.6±5.6 | 0.017 |
| *Pre-TAVR dPmean - mmHg* | 43.5±15.8 | 44.6±16.5 | 46.3±18.0 | **<0.001** |
| *Pre-TAVR LVEF - %* | 57.6±10.8 | 56.5±11.4 | 54.4±13.1 | **<0.001** |
| *NYHA class ≥ III* | 5602 (74.5%) | 1412 (73.7%) | 304 (77.4%) | 0.304 |
| *Annulus area - mm^2^* | 410±64.4 | 406±63.3 | 395±70.0 | **<0.001** |
| *Annulus area/height - mm^2^/m* | 2.56±0.39 | 2.49±0.37 | 2.41±0.41 | **<0.001** |
| *Self-expanding device* | 5355 (71.0%) | 1045 (54.3%) | 158 (40%) | **<0.001** |
| *Procedure: pre-dilation* | 4674 (62.8%) | 981 (52.3%) | 187 (51.8%) | **<0.001** |
| *Procedure: post-dilation* | 1941 (26.0%) | 367 (19.6%) | 61 (16.9%) | **<0.001** |
| *EOA – cm²* | 1.90±0.41 | 1.32±0.14 | 1.02±0.15 | **<0.001** |
| *EOAi – cm²/m²* | 1.10±0.25 | 0.74±0.08 | 0.57±0.07 | **<0.001** |
| *PVL ≥ moderate* | 224 (3.5%) | 71 (4.1%) | 11 (3.1%) | 0.426 |
| *Comorbidities* |  |  |  |  |
| *GFR - ml/min* | 60.2±21.3 | 59.4±21.1 | 60.1±21.7 | 0.285 |
| *Hypertension* | 6254 (90.4%) | 1639 (91.8%) | 337 (89.4%) | 0.137 |
| *COPD* | 658 (13.3%) | 195 (13.2%) | 37 (12.9%) | 0.979 |
| *CAD* | 3537 (46.9%) | 928 (48.3%) | 196 (49.6%) | 0.355 |
| *Diabetes* | 2350 (31.4%) | 620 (32.5%) | 129 (33.1%) | 0.542 |
| *Hyperlipidemia* | 2150 (58.4%) | 650 (53.0%) | 135 (55.1%) | **0.004** |
| *Atrial fibrillation/flutter* | 2507 (33.3%) | 736 (38.4%) | 156 (39.7%) | **<0.001** |
| *Prior stroke/TIA* | 1416 (19.2%) | 392 (21.2%) | 77 (19.9%) | 0.173 |
| *Prior cardiac surgery* | 391 (5.2%) | 140 (7.3%) | 46 (11.6%) | **<0.001** |
| *Prior pacemaker* | 654 (8.7%) | 195 (10.2%) | 36 (9.1%) | 0.131 |

Baseline characteristics of the female patient cohort considering PPM severity are shown. Continuous values are presented as mean±SD, categorical values as n (%).

Abbreviations: BMI: body mass index; BSA: body surface area; CAD: coronary artery disease; COPD: chronic obstructive pulmonary disease; dPmean; mean transvalvular pressure gradient; GFR: glomerular filtration rate; LVEF: left ventricular ejection fraction; PPM: prosthesis-patient mismatch; PVL: paravalvular leak; SD: standard deviation; STS: Society of Thoracic Surgeons; TIA: transient ischemic attack.

**Supplementary Table 3: Characteristics of the Male Patient Cohort**

| **Parameter** | **PPM:**  **no** | **PPM:**  **moderate** | **PPM:**  **severe** | ***P* value** |
| --- | --- | --- | --- | --- |
| *N* | 7537 | 2236 | 461 |  |
| *Age - years* | 80.4±6.2 | 79.8±6.6 | 78.9±7.3 | **<0.001** |
| *BMI - kg/m^2^* | 27.5±4.6 | 27.4±4.4 | 27.7±4.9 | 0.770 |
| *BSA - m^2^* | 1.95±0.18 | 1.99±0.18 | 2.03±0.19 | **<0.001** |
| *STS Score - %* | 4.6±4.1 | 4.6±4.4 | 4.9±4.4 | 0.305 |
| *Pre-TAVR dPmean - mmHg* | 41.2±14.1 | 41.9±14.7 | 38.8±13.6 | **<0.001** |
| *Pre-TAVR LVEF - %* | 53.2±12.7 | 51.5±13.3 | 48.3±14.0 | **<0.001** |
| *NYHA class ≥ III* | 5081 (67.7%) | 1493 (67.0%) | 312 (70.3%) | 0.390 |
| *Annulus area - mm^2^* | 511±81 | 521±85 | 519±79 | **<0.001** |
| *Annulus area/height - mm^2^/m* | 2.96±0.45 | 2.97±0.46 | 2.92±0.42 | 0.399 |
| *Self-expanding device* | 3829 (50.8%) | 720 (32.2%) | 129 (28.0%) | **<0.001** |
| *Procedure: pre-dilation* | 4171 (56.2%) | 928 (42.1%) | 152 (34.2%) | **<0.001** |
| *Procedure: post-dilation* | 1768 (23.7%) | 363 (16.4%) | 62 (13.9%) | **<0.001** |
| *EOA – cm²* | 2.11±0.44 | 1.48±0.15 | 1.16±0.16 | **<0.001** |
| *EOAi – cm²/m²* | 1.09±0.24 | 0.75±0.07 | 0.57±0.07 | **<0.001** |
| *PVL ≥ moderate* | 229 (3.5%) | 66 (3.2%) | 7 (1.6%) | 0.106 |
| *Comorbidities* |  |  |  |  |
| *GFR - ml/min* | 64.2±21.4 | 64.0±22.1 | 60.8±22.2 | **0.005** |
| *Hypertension* | 6246 (90.1%) | 1831 (88.5%) | 387 (88.2%) | 0.075 |
| *COPD* | 777 (15.4%) | 297 (16.7%) | 53 (14.1%) | 0.291 |
| *CAD* | 5013 (66.5%) | 1517 (67.9%) | 302 (65.5%) | 0.402 |
| *Diabetes* | 2593 (34.9%) | 750 (34.0%) | 170 (37.9%) | 0.280 |
| *Hyperlipidemia* | 2392 (66.1%) | 909 (61.6%) | 194 (61.6%) | **0.005** |
| *Atrial fibrillation/flutter* | 2804 (37.2%) | 958 (42.9%) | 241 (52.5%) | **<0.001** |
| *Prior stroke/TIA* | 1566 (21.2%) | 481 (22.4%) | 106 (23.8%) | 0.246 |
| *Prior cardiac surgery* | 1040 (13.8%) | 323 (14.4%) | 78 (16.9%) | 0.149 |
| *Prior pacemaker* | 1026 (13.6%) | 310 (13.9%) | 90 (19.5%) | **0.002** |

Baseline characteristics of the male patient cohort considering PPM severity are shown. Continuous values are presented as mean±SD, categorical values as n (%).

Abbreviations: BMI: body mass index; BSA: body surface area; CAD: coronary artery disease; COPD: chronic obstructive pulmonary disease; dPmean; mean transvalvular pressure gradient; GFR: glomerular filtration rate; LVEF: left ventricular ejection fraction; PPM: prosthesis-patient mismatch; PVL: paravalvular leak; SD: standard deviation; STS: Society of Thoracic Surgeons; TIA: transient ischemic attack.

**Supplementary Table 4: Sex-specific Differences in Patients with Severe PPM**

| **Parameter** | **Severe PPM**  ***Female*** | **Severe PPM**  ***Male*** | ***P* value** |
| --- | --- | --- | --- |
| *N* | 395 | 461 |  |
| *Age - years* | 80.2±7.0 | 78.9±7.3 | **0.006** |
| *BMI - kg/m^2^* | 27.7±5.9 | 27.7±4.9 | 0.495 |
| *BSA - m^2^* | 1.80±0.20 | 2.03±0.19 | **<0.001** |
| *STS Score - %* | 6.6±5.6 | 4.9±4.4 | **<0.001** |
| *Pre-TAVR dPmean - mmHg* | 46.3±18.0 | 38.8±13.6 | **<0.001** |
| *Pre-TAVR LVEF - %* | 54.4±13.1 | 48.3±14.0 | **<0.001** |
| *NYHA class ≥ III* | 304 (77.4%) | 312 (70.3%) | **0.024** |
| *Annulus area - mm^2^* | 395±70.0 | 519±79 | **<0.001** |
| *Annulus area/height - mm^2^/m* | 2.41±0.41 | 2.92±0.42 | **<0.001** |
| *Self-expanding device* | 158 (40.0%) | 129 (28.0%) | **<0.001** |
| *Procedure: pre-dilation* | 187 (51.8%) | 152 (34.2%) | **<0.001** |
| *Procedure: post-dilation* | 61 (16.9%) | 62 (13.9%) | 0.239 |
| *EOA – cm²* | 1.02±0.15 | 1.16±0.16 | **<0.001** |
| *EOAi – cm²/m²* | 0.57±0.07 | 0.57±0.07 | **0.044** |
| *PVL ≥ moderate* | 11 (3.1%) | 7 (1.6%) | 0.232 |
| *Comorbidities* |  |  |  |
| *GFR - ml/min* | 60.1±21.7 | 60.8±22.2 | 0.534 |
| *Hypertension* | 337 (89.4%) | 387 (88.2%) | 0.657 |
| *COPD* | 37 (12.9%) | 53 (14.1%) | 0.732 |
| *CAD* | 196 (49.6%) | 302 (65.5%) | **<0.001** |
| *Diabetes* | 129 (33.1%) | 170 (37.9%) | 0.170 |
| *Hyperlipidemia* | 135 (55.1%) | 194 (61.6%) | 0.141 |
| *Atrial fibrillation/flutter* | 156 (39.7%) | 241 (52.5%) | **<0.001** |
| *Prior stroke/TIA* | 77 (19.9%) | 106 (23.8%) | 0.208 |
| *Prior cardiac surgery* | 46 (11.6%) | 78 (16.9%) | **0.032** |
| *Prior pacemaker* | 36 (9.1%) | 90 (19.5%) | **<0.001** |

Baseline characteristics of female and male patients with severe PPM severity are shown. Continuous values are presented as mean±SD, categorical values as n (%).

Abbreviations: BMI: body mass index; BSA: body surface area; CAD: coronary artery disease; COPD: chronic obstructive pulmonary disease; dPmean; mean transvalvular pressure gradient; GFR: glomerular filtration rate; LVEF: left ventricular ejection fraction; PPM: prosthesis-patient mismatch; PVL: paravalvular leak; SD: standard deviation; STS: Society of Thoracic Surgeons; TIA: transient ischemic attack.

**Supplementary Figure 1: Post-TAVR dPmean by Device Type**

Stratified by device type, dPmean was slightly higher in females in both BE and SE devices.

Abbreviations: BE: balloon-expandable; dPmean: mean transvalvular pressure gradient; SE: self-expanding.

**IMPPACT TAVR Investigators**

Ahmed Abdelhafez (Leipzig), Ignacio J. Amat-Santos (Valladolid), Dabit Arzamendi (Barcelona), Marco Barbanti (Enna), Luca Branca (Brescia), Matjaz Bunc (Ljubljana), Jonathan Curio (Cologne), Ole De Backer (Copenhagen), Rodrigo Estévez-Loureiro (Vigo), Ariel Finkelstein (Tel Aviv), Christian Frerker (Lübeck), Salome Hecht (Leipzig), Manuel Hein (Freiburg), Michael Joner (Munich), Malte Kelm (Düsseldorf), Samuel Lee (Cologne), Valerie Lohner (Cologne), Sebastian Ludwig (Hamburg), Max M Meertens (Cologne), Helge Möllmann (Dortmund), Ute Mons (Cologne), Darren Mylotte (Galway), Georg Nickenig (Bonn), Luis Nombela-Franco (Madrid), Markus Pauly (Dortmund), Costanza Pellegrini (Munich), Max Potratz (Bad Oeynhausen), Andreas Rück (Stockholm), Philipp Ruile (Freiburg), Corrado Tamburino (Catania), Giuseppe Tarantini (Padova), Maria Thurow (Dortmund)
